# p53 and VEGF expression are independent predictors of tumour recurrence and survival following curative resection of gastric cancer

**DOI:** 10.1038/sj.bjc.6601455

**Published:** 2004-01-06

**Authors:** C Fondevila, J P Metges, J Fuster, J J Grau, A Palacín, A Castells, A Volant, M Pera

**Affiliations:** 1 Services of Gastrointestinal Surgery, Institut de Malalties Digestives, Hospital Clínic, Institut d'Investigació Biomèdica August Pi i Sunyer (IDIBAPS), University of Barcelona Medical School, Barcelona, Spain; 2 Departments of Pathology and Medical Oncology, Centre Hospitalier Universitaire Cavale Blanche and Morvan, Brest, France; 3 Department of Medical Oncology, Hospital Clínic, IDIBAPS, University of Barcelona Medical School, Barcelona, Spain; 4 Department of Pathology, Hospital Clínic, IDIBAPS, University of Barcelona Medical School, Barcelona, Spain; 5 Services of Gastroenterology, Institut de Malalties Digestives, Hospital Clínic, Institut d'Investigació Biomèdica August Pi i Sunyer (IDIBAPS), University of Barcelona Medical School, Barcelona, Spain

**Keywords:** angiogenesis, gastric cancer, p53, VEGF, prognostic factors, microvessel density

## Abstract

This study was undertaken to determine the value of tumour microvessel density (MVD) and the expression of p53 and vascular endothelial growth factor (VEGF) as prognostic markers in patients with gastric cancer operated on for cure. In all, 156 patients with curatively resected gastric cancer constituted the basis of this blinded retrospective evaluation. Patients were treated with either surgery alone (*n*=53) or surgery plus adjuvant chemotherapy (*n*=103). Tumour MVD, p53 expression, and VEGF expression were assayed using immunohistochemical techniques. After a mean follow-up of 43 months, 64 (41%) patients had died and 55 (35%) patients developed tumour recurrence. Positive correlations between MVD and both p53 (*P*=0.005) and VEGF (*P*=0.005) expression were observed. Both MVD ⩾100 (*P*=0.05) and positive VEGF expression (*P*<0.02) were associated with shorter disease-free survival, and positive VEGF expression (*P*=0.01) was also associated with shorter overall survival. Multivariate analysis confirmed that, in addition to the pathological tumour stage, lymph node ratio, the extent of lymphadenectomy and perineural invasion, p53 expression, and VEGF expression were independently associated with both disease-free survival (*P*<0.0005 and 0.02, respectively) and overall survival (*P*<0.02 and 0.01, respectively). Finally, patients whose tumours did not show p53 expression had a survival benefit compared to those expressing p53 when treated with adjuvant chemotherapy (*P*=0.01).This investigation demonstrates that p53 expression and VEGF expression are independent prognostic factors for both disease-free survival and overall survival in patients with curatively resected gastric cancer, and that p53 status may also influence response to chemotherapy.

Until the 1980s, gastric cancer was one of the most frequent tumours in the world and the leading cause of cancer death (Parkin *et al*, 1999). In recent decades, the incidence has declined, but the prognosis of gastric cancer in the Western countries has not improved, the 5-year survival being 20–30% (Hundahl *et al*, 2000; Greenlee *et al*, 2001). Surgical resection is the most powerful tool to improve prognosis, whereas the major problem is delayed diagnosis resulting in advanced disease. In most American and European series, almost 60% of operated patients have pathological tumour (pT) stages 3 and 4. Screening programmes are not usually performed due to their high cost and minimal benefit in decreasing mortality rates (Lacueva and Calpena, 2001).

In curatively resected patients, the biologic nature of the tumour determines survival since almost half will die from recurrent cancer (Averbach and Jacquet, 1996). The poor prognosis of patients with recurrent disease is due to the lack of an effective rescue treatment. In fact, the number of patients with recurrent gastric cancer in which it is feasible to perform curative surgery is less than 4% (Yoo *et al*, 2000). It seems necessary to evaluate new biological markers that may predict the natural history of the disease as a guide to treatment. Newer tumour markers include tumour suppressor gene p53, vascular endothelial growth factors (VEGFs), and microvessel density (MVD) as a measure of new blood vessel growth or angiogenesis. Without blood vessels, tumours cannot grow beyond a critical mass nor create metastases (Castells and Rustgi, 2003). A hypoxic environment and genetic instability in the centre of the tumour allows the evolution of cellular clones with the loss of p53 function. These cells have a lower apoptotic rate and produce angiogenic factors like VEGF, inducing new vasculature (Harris, 1997). These factors might provide information to help predict the prognosis of patients with gastric cancer. However, the prognostic value of p53 expression in these patients is controversial (Gabbert *et al*, 1995), although it has been related to the development of higher MVD (Maehara *et al*, 2000b). The expression of VEGF has been associated with vascular invasion, liver metastases (Takahashi *et al*, 1996), and lymph node metastases (Maeda *et al*, 1996). Inhibition of the VEGF pathway using monoclonal antibodies has shown potent antitumour effects in animal models (Witte *et al*, 1998), and this might be a new approach to treat these patients in the future.

The use of adjuvant chemotherapy in gastric cancer is still controversial and has been reviewed in several meta-analyses (Hermans *et al*, 1993; Earle and Maroun, 1999). One review concluded that adjuvant chemotherapy produced marginal benefit in the survival of curatively resected patients (Earle and Maroun, 1999), but it is clearly necessary to find more effective treatments. Differing responses to chemotherapy may be due to biological characteristics of the tumour such as p53 status, since p53-dependent apoptosis modulates the cytotoxic effects of antitumour agents such as 5-fluorouracil, doxorubicin, and cisplatin (Chin *et al*, 1992; Lowe *et al*, 1993), commonly used as adjuvant therapy. Knowing the p53 status of the individual patient might allow us to select those cases most likely to respond to adjuvant therapy.

The aim of this study was (1) to investigate whether MVD as well as p53 expression and VEGF expression are independent prognostic factors for patients with gastric cancer undergoing curative gastrectomy, and (2) to evaluate their predictive value for clinical outcome following adjuvant chemotherapy.

## PATIENTS AND METHODS

A consecutive series of 206 patients with primary gastric cancer received surgical treatment at our hospital between January 1989 and October 1998. The mean age was 67±12 years (range: 23–93 years), and 130 (63%) patients were male. Of them, 164 (80%) patients underwent potentially curative resection (R0 resection following UICC criteria^18^), defined as macroscopically and microscopically complete removal of the tumour on intraoperative and histopathologic evaluation. Eight patients (5%) died within 60 days after surgical treatment and were excluded from this analysis. Accordingly, 156 patients constituted the basis of the study.

The characteristics of patients included in the study are shown in Table 1

**Table 1:** Demographic, clinical, and tumor characteristics of patients included in the study (*n*=156)

*Age (years)*	
<70	88 (56%)
⩾70	68 (44%)

*Gender*	
Male	101 (65%)
Female	55 (35%)

*Tumour location*	
Cardiac fundus	11 (7%)
Body	62 (40%)
Antrum pylorus	80 (51%)
Diffuse	3 (2%)

*Lauren classification*	
Intestinal	97 (62%)
Diffuse	59 (38%)
Tumour size (mm)	54±27

*Degree of differentiation*	
Well	6 (4%)
Moderate	73 (47%)
Poor	77 (49%)

*Signet-ring cell-type tumours*	41 (26%)

*pT stage*^a^	
T1	28 (18%)
T2	75 (48%)
T3	51 (33%)
T4	2 (1%)

*pN stage*^a^	
N0	66 (42%)
N1	54 (35%)
N2	33 (21%)
N3	3 (2%)

*Lymph-node ratio*^b^	
0	66 (42.3%)
<30%	48 (30.8%)
⩾30%	42 (26.9%)
Lymphatic invasion	44 (28.2%)
Vascular invasion	17 (10.9%)
Perineural invasion	20 (12.8%)

*pM stage*^a^	
M0	151 (96.8%)
M1	5 (3.2%)

*pTNM tumor stage*^a^	
IA	19 (12.2%)
IB	39 (25%)
II	42 (26.9%)
IIIA	27 (17.3%)
IIIB	21 (13.5%)
IV	8 (5.1%)

a According to the 5th edition of the tumour-node metastasis classification of the International Union Against Cancer (UICC)^18^.

b Number of affected lymph nodes divided by the total number of lymph nodes.

pT=pathological tumour; pTNM=pathological tumour node metastasis. pN=pathological lymph node; pM=pathological metastasis.

. Staging and grading were referred to the fifth edition of the tumour-node metastasis (TNM) classification of the International Union Against Cancer (UICC) (Sobin and Fleming, 1997). Accordingly, 19 patients (12%) were classified as UICC stage IA, 39 (25%) as stage IB, 42 (27%) as stage II, 27 (17%) as stage IIIA, 21 (13%) as stage IIIB, and eight (5%) as stage IV.

The surgical technique in this group was as follows: 76 subtotal gastrectomies (49%), 77 total gastrectomies (49%), and three stump gastrectomies (2%). Based on the decision of the surgeon, 50 (32%) patients had a D1 lymphadenectomy, including the first-level lymph nodes (paracardial, major and minor curvature, supra-, and infrapyloric) and 106 (68%) patients had a D2 lymphadenectomy, in which the second-level nodes (left gastric artery, hepatic artery, celiac trunk, splenic hilum, and splenic artery) were also excised. Splenectomy to ensure complete resection of the tumour was performed in 49 patients (31%). A perioperative histological frozen section of the resection margin was always performed to rule out tumour invasion.

In all, 103 patients (66%) received adjuvant chemotherapy based on either mitomycin C (10–20 mg/ m^2^ i.v. every 6 weeks) (*n*=38) or mitomycin C (10–20 mg/ m^2^ i.v. on day 1 every 6 weeks) plus tegafur (500 mg /m^2^ day^1^ for 36 consecutive days) (*n*=65). The courses were repeated four times as reported previously (Grau *et al*, 1998).

### Follow-up

Patients were followed up at 3-month intervals over the first 2 years, and at 6-month intervals thereafter. Medical work-up consisted of history and physical examination, haematology and biochemical tests, including serum carcinoembryonic antigen, carbohydrate antigen 19-9, and tissue-associated glycoprotein 72-4 concentration, and chest radiography, abdominal ultrasonography, and endoscopy. If local tumour recurrence and/or metastasis were suspected, the confirmation of the diagnosis by biopsy or a second surgical exploration was attempted.

### Immunohistochemical methods

Paraffin-embedded tissue blocks of formalin-fixed surgically resected samples were processed for conventional histological study and for immunohistochemical analysis. Immunohistochemical studies were performed using the automated immunohistochemical system TechMate 500 (Dako, Carpinteria, CA, USA), using the EnVision system (Dako). Briefly, 4 *μ*m-thick sections were deparaffinised and hydrated through graded alcohol and water. Peroxidase was blocked for 7.5 min in ChemMate peroxidase-blocking solution (Dako). Then the slides were incubated with the primary antibodies for 30 min and washed in ChemMate buffer solution (Dako). The peroxidase-labelled polymer was then applied for 30 min. After washing in ChemMate buffer solution, the slides were incubated with the diaminobenzidine substrate chromogen solution, washed in water, counterstained with haematoxylin, washed, dehydrated, and mounted. Antigen retrieval was performed in citrate buffer, pH 6.0, in a pressure cooker. The following antibodies were used in this study: monoclonal antibody against p53 (clone BP53-12 at a 1 : 50 dilution, Novocastra, Newcastle upon Tyne, UK), polyclonal antibody against VEGF (clone A-20 at a 1 : 300 dilution, Santa Cruz Biotechnology, Santa Cruz, CA, USA), and a monoclonal antibody against the CD34 antigen (clone QBEnd/10 at a 1 : 200 dilution; Novocastra, Newcastle upon Tyne, UK).

#### p53 staining analysis

**Figure 1:**
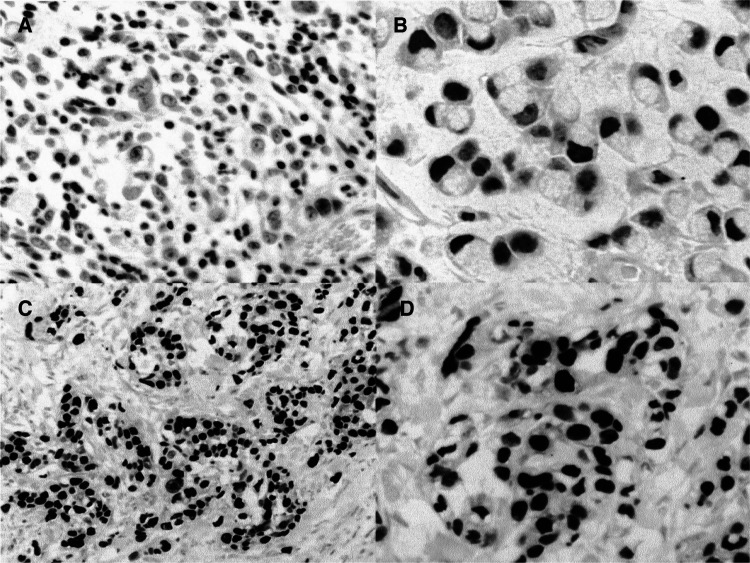
Diffuse-type gastric cancer with signet ring nucleus. (**A** and **B**) No expression of p53. (**C** and **D**) Intense staining in the irregular nucleus of tumour cells.

Using a light microscope, a visual grading system based on the number of positively stained nuclei of the malignant cells in each tissue was used (Figure 1

). If 10% or more of the malignant nuclei were stained, the slide was scored as positive. If fewer than 10% of the nuclei were stained, the slide was scored as negative, in accordance with other authors (Kakeji *et al*, 1993; Maehara *et al*, 1999). All specimens were analysed by two separate investigators (CF and AV) who were blinded to all clinical information. Conflicts in scores were resolved by consensus.

#### VEGF staining analysis

**Figure 2:**
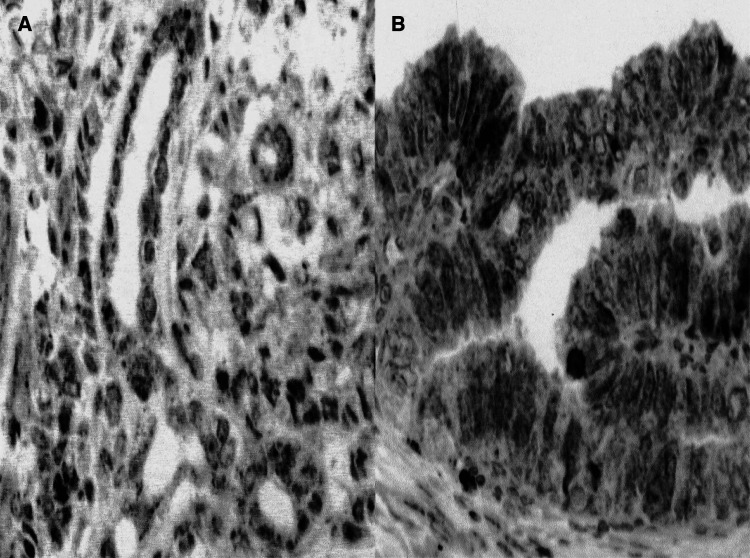
Two samples of gastric tumours with expression of VEGF: **A** (+), **B** (++). The VEGF appears in the cytoplasm of cells. In (**B**) the intensity is higher than in (**A**). The evaluation was carried out in the tumour margins far from the tumour centre where the hypoxia can induce VEGF expression.

Vascular endothelial growth factor immunostaining was considered to be positive when unequivocal cytoplasmic staining was seen in the tumour cells, regardless of the number of cells stained. Vascular endothelial growth factor expression was analysed in the invasive front of the tumour away from the tumour centre where necrosis and hypoxia may induce VEGF expression. The intensity of staining for VEGF was graded as follows: −, no detectable expression; +, moderate stain; ++, strongest stain under a × 250 field (Figure 2

). As described by Inoue *et al* (1997), we used smooth muscle cells as a positive internal control. Two different investigators (CF and AV) assessed the degree of staining without knowledge of the clinical data.

#### Microvessel staining and counting

**Figure 3:**
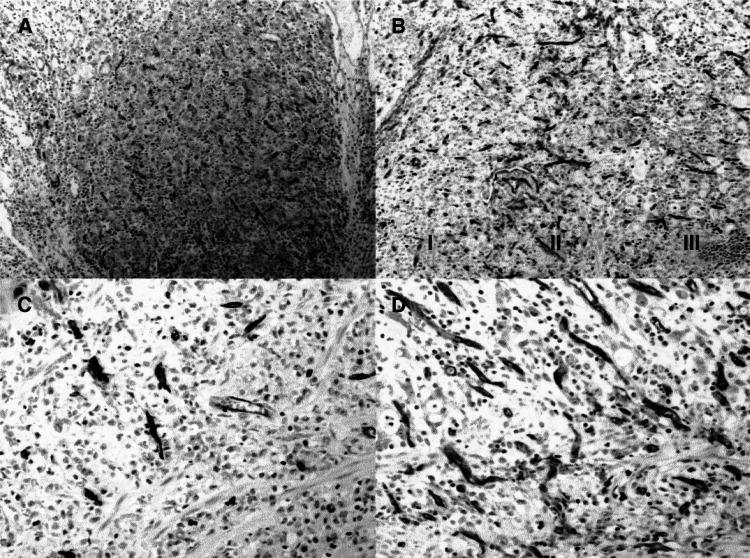
Evaluation of tumour angiogenesis. Identification of external border of tumour growth at × 40 (**A**) and × 100 (**B**) magnification field: I (desmoplastic stroma), II (infiltration zone), and III (tumour). At × 250 magnification field (**C** and **D**), it is possible to appreciate the differences in the degree of MVD between tumours.

Quantitative vessel counts were performed by the method described by Weidner and assessed by international consensus (Vermeulen *et al*, 1996). The entire tumour sections were systematically scanned at × 40 magnification to find the areas of most intense neovascularisation or hot spots. These were identified as having the highest density of brown staining, CD34-positive cells, or cell clusters. For each slide, the most vascular areas within the tumour mass were chosen (Figure 3

). A × 250 field in these areas was counted, and the average counts of the fields were recorded. If multiple vascular hot spots were present, counts were performed in each hot spot. Microvessels were defined as a discrete CD34-positive endothelial cell aggregate, with or without definable lumina. The microvessels were counted by two investigators (CF and AV) who had no knowledge of the other prognostic factors and/or clinical outcomes.

### Statistical methods

The relationship between tumour MVD, p53 expression, and VEGF expression, as well as their correlation with clinicopathological parameters were evaluated by the *χ*^2^ test. The length of follow-up was described as the mean, 95% confidence interval (95% CI), and range. The impact of single parameters on prognosis (tumour recurrence, disease-free survival, and overall survival) was determined by both univariate and multivariate approaches. Tumour recurrence was evaluated by means of logistic regression analysis. Probabilities of disease-free survival and overall survival were calculated according to the Kaplan–Meier method and compared with the log-rank test. Variables achieving a significance level of *P*⩽0.1 in the univariate analysis were subsequently introduced in a forward stepwise proportional-hazard analysis (Cox's model) to identify those variables independently associated with survival. Disease-free survival was established from the time of surgery to the date when recurrence or death was detected. Overall survival was established from the date of surgery to death from any cause or the date of the last follow-up visit. In both cases, patients who were event free at the end of follow-up were censored at that time. For continuous variables (i.e. MVD and lymph node ratio), the cut-off level chosen was their median value. Vascular endothelial growth factor categories were reclassified as negative and positive (including moderate and strong staining) for statistical purposes. Variables actually reflecting a combination of independent parameters (i.e. TNM stage) were not included in the multivariate analysis as a single covariable, but rather decomposed in the corresponding original counterparts (T, N, and M components). All statistical analyses were performed two-sided at a significance level of *P*=0.05, using the statistical package SPSS (SPSS Inc., Chicago, IL, USA).

## RESULTS

After a mean follow-up of 43 months (95% CI: 37–49 months; range: 2–191 months), 64 (41%) patients had died, the probability of survival being 69 and 54% at 2 and 5 years, respectively. A total of 50 patients died because of tumour progression and 14 due to other causes. In all, 55 (35%) patients developed tumour recurrence, 33 of whom presented as locoregional relapse, 12 as distant metastases (eight in liver, three in lung, and one in bone), nine as peritoneal seeding, and one as stump. All but three patients with tumour recurrence died from it.

### Correlation between MVD, p53 expression, and VEGF expression

The median microvessel count was 98.9. (95% CI: 94.7–103.2), and this value was used to dichotomise the series. p53 expression was detected in 71 (46%) patients (Table 2

**Table 2:** Distribution of tumour microvessel density, p53 expression, and VEGF expression in patients included in the study (*n*=156)

*Tumor microvessel density*	
<100	74 (47.4%)
⩾100	82 (52.6%)

*p53 expression*	
Negative	85 (54.5%)
Positive	71 (45.5%)

*VEGF expression*	
Negative	40 (25.6%)
Positive+	83 (53.2%)
Positive++	33 (21.2%)

VEGF=vascular endothelial growth factor.

). p53 protein expression was significantly associated with MVD ⩾100 (OR: 2.22, 95% CI: 1.26–3.88; *P*=0.005).

Vascular endothelial growth factor expression was detected in 116 (74%) patients (Table 2). There was a significant association with MVD ⩾100 (OR: 2.65, 95% CI: 1.33–5.29; *P*=0.005). There was no association between p53 and VEGF expression.

### Correlation between MVD, p53 expression, and VEGF expression, and clinicopathological characteristics

Neither p53 positivity nor VEGF expression was related to any of the clinicopathological parameters reported in Table 1. On the contrary, a statistically significant association was found between MVD ⩾100 and lymph node metastases (*P*<0.003). As shown in Table 3

**Table 3:** Correlation between tumour microvessel density and lymph node metastases

	**Tumor microvessel density**		
	**<100 (*n*=74)**	**⩾100 (*n*=82)**	***P*-value^a^**
*Lymph node ratio*^b^			
0% (*n*=66)	40	26	
<30% (*n*=48)	18	30	<0.02
⩾30% (*n*=42)	16	26	

*pN stage*^c^			
N0 (*n*=66)	40	26	
N1 (*n*=54)	19	35	<0.003
N2 (*n*=33)	13	20	
N3 (*n*=3)	2	1	

a *χ*^2^ test.

b Number of affected lymph nodes divided by the total number of lymph nodes.

c According to the 5th edition of the tumour-node-metastasis classification of the International Union Against Cancer (UICC)^18^; pN=pathological lymph node.

, the lymph node ratio was also higher when MVD was elevated (*P*<0.02), as well as the pN stage (*P*<0.003).

### Tumour recurrence and disease-free survival

There were statistically significant associations between tumour recurrence and tumour MVD ⩾100, p53 expression, and VEGF expression (Table 4

**Table 4:** Influence of tumour microvessel density, p53 expression, and VEGF expression on tumor recurrence and disease-free survival (univariate analysis)

	**Tumour recurrence^a^**					**Disease-free survival^b^**
	**No**	**Yes**	***P*-value**	**OR**	**95% CI**	***P*-value**
*Microvessel density*						
<100	55 (74%)	19 (26%)		1^c^		
⩾100	46 (56%)	36 (44%)	<0.02	2.26	1.15–4.47	0.05

*p53 expression*						
Negative	61 (72%)	24 (28%)		1^c^		
Positive	40 (56%)	31 (44%)	<0.05	1.96	1.01–3.83	0.09

*VEGF expression*						
Negative	32 (80%)	8 (20%)		1^c^		
Positive	69 (59%)	47 (40%)	<0.02	2.72	1.15–6.43	<0.02

a Logistic regression; OR=odds ratio; 95% CI=95% confidence interval; VEGF=vascular endothelial growth factor.

b Log-rank test.

c Reference category.

). Other significant parameters found in the univariate analysis of tumour recurrence were pT stage (*P*<0.0001), pN stage (*P*<0.0001), pTNM stage (*P*<0.0001), lymph node ratio (*P*<0.0001), lymphatic invasion (*P*<0.0001), Lauren classification (*P*<0.005), signet-ring cell-type tumours (*P*<0.013), degree of differentiation (*P*<0.008), and extent of lymphadenectomy (*P*<0.0001).

Both MVD ⩾100 (*P*=0.05) and VEGF expression (*P*<0.02) were associated with a shorter disease-free survival in the univariate analysis (Table 4). Other significant parameters found in the analysis of disease-free survival were pT stage (*P*<0.0001), pN stage (*P*<0.0001), pTNM stage (*P*<0.0001), lymph node ratio (*P*<0.0001), lymphatic invasion (*P*<0.0001), Lauren classification (*P*<0.0039), signet-ring cell-type tumours (*P*<0.01), degree of differentiation (*P*<0.0048), and extent of lymphadenectomy (*P*<0.0001). No correlation between MVD, p53 expression, and VEGF expression, and the type of recurrence (locoregional relapse *vs* distant metastases) was found.

The multivariate analysis identified lymph node ratio ⩾30%, D1 lymphadenectomy, p53 expression, and VEGF expression as independent predictors of tumour recurrence and disease-free survival (Table 5

**Table 5:** Prognostic factors of tumour recurrence and disease-free survival (multivariate analysis)

	**Tumor recurrence^a^**					**Disease-free survival^b^**
	**No**	**Yes**	***P*-value**	**OR**	**95% CI**	***P*-value**
*Lymphadenectomy*						
D2	80 (75%)	26 (24%)		1^c^		
D1	21 (42%)	29 (58%)	0.0005	6.93	2.33–20.55	<0.0001

*Lymph node ratio*						
0	55 (83%)	11 (17%)		1^c^		
<30%	32 (67%)	16 (33%)	0.06	3.11	0.95–10.16	0.13
⩾30%	14 (33%)	28 (67%)	<0.001	9.98	2.57–38.76	<0.005

*p53 expression*						
Negative	61 (72%)	24 (28%)		1^c^		
Positive	40 (56%)	31 (44%)	<0.02	3.36	1.29–8.74	<0.0005

*VEGF expression*						
Negative	32 (80%)	8 (20%)		1^c^		
Positive	69 (59%)	47 (40%)	<0.005	5.88	1.71–20.26	<0.02

a Logistic regression; OR=odds ratio; 95% CI=95% confidence interval; VEGF=vascular endothelial growth factor.

b Cox's regression.

c Reference category.

).

### Overall survival

**Figure 4:**
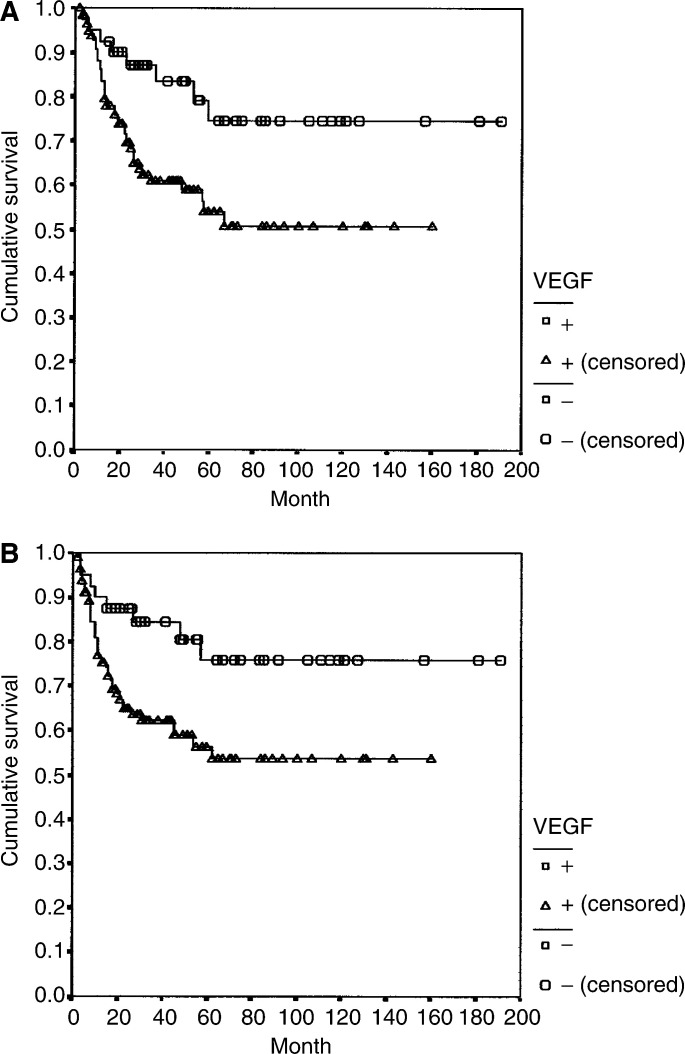
(**A**) Overall survival in patients with VEGF-negative and VEGF-positive tumours (*P*<0.02). (**B**) Disease-free survival in patients with VEGF-negative and VEGF-positive tumours (*P*<0.02).

In the univariate analysis, factors influencing overall survival were pTNM stage (*P*<0.0001), pT (*P*<0.0001), pN (*P*<0.0001), lymph node ratio (*P*<0.0001), signet-ring cell-type tumours (*P*<0.01), degree of differentiation (*P*<0.005), Lauren classification (*P*<0.005), lymphatic invasion (*P*<0.0001), perineural invasion (*P*<0.39), and extent of lymphadenectomy (*P*<0.0001). In addition, VEGF expression was associated with a shorter overall survival (HR: 2.48, 95% CI: 1.16–5.28; *P*=0.01) (Figure 4

). By contrast, neither p53 expression (HR: 1.61, 95% CI: 0.93–2.80; *P*=0.08) nor MVD ⩾100 (HR: 1.70, 95% CI: 0.95–3.02; *P*=0.07) reached statistical significance in this analysis.

The multivariate Cox's regression analysis revealed that pT3 stage or higher, lymph node ratio ⩾30%, D1 lymphadenectomy, the presence of perineural invasion, p53 expression, and VEGF expression were independently associated with a shorter overall survival (Table 6

**Table 6:** Prognostic factors of overall survival (multivariate analysis)

	**Dead**	**Alive**	**HR**	**95% CI**	***P*-value**
*pT stage*^b^					
T1	4 (14%)	24 (86%)	1^c^		
T2	16 (21%)	59 (79%)	1.18	0.37–3.78	0.77
T3	31 (58%)	22 (42%)	3.74	1.16–12.02	<0.03

*Lymph node ratio*^d^					
0	10 (15%)	56 (85%)	1^c^		
<30%	14 (29%)	34 (71%)	2.95	1.14–7.66	<0.03
⩾30%	27 (64%)	15 (36%)	7.75	3.25–18.47	<0.0001

*Lymphadenectomy*					
D2	24 (23%)	82 (77%)	1^c^		
D1	27 (54%)	23 (46%)	4.40	2.30–8.44	<0.0001

*Perineural invasion*					
Absence	44 (32%)	92 (68%)	1^c^		
Presence	7 (35%)	13 (65%)	2.83	1.17–6.85	<0.03

*p53 expression*					
Negative	23 (27%)	62 (73%)	1^c^		
Positive	28 (39%)	43 (60%)	2.30	1.21–4.36	<0.02

*VEGF expression*					
Negative	8 (20%)	32 (80%)	1^c^		
Positive	43 (37%)	73 (63%)	2.99	1.34–6.67	<0.01

a Cox's regression; HR=hazard ratio; 95 %CI=95% confidence interval; VEGF=vascular endothelial growth factor; pT=pathological tumour.

b According to the 5th edition of the tumor-node metastasis classification of the International Union Against Cancer (UICC)^18^.

c Reference category.

d Number of affected lymph nodes divided by the total number of lymph nodes.

).

### Influence of MVD, p53 expression, and VEGF expression on the clinical outcome of patients receiving chemotherapy

The predictive value of MVD, p53 expression, and VEGF expression was also evaluated in the subset of 103 patients who received adjuvant chemotherapy. As shown in Table 7

**Table 7:** Influence of tumour microvessel density, p53 expression, and VEGF expression on overall survival following adjuvant chemotherapy

	**Patients receiving chemotherapy(*n*=103)**			**Patients not receiving chemotherapy (*n*=53)**		
	***n***	**Survival (%)^a^**	**P-value**	***n***	**Survival (%)^a^**	**P-value**
*Microvessel density*						
<100	45	76	0.58	29	31	
⩾100	58	71		24	33	0.86

*p53 expression*						
Negative	58	83	0.01	27	30	0.69
Positive	45	60		26	35	

*VEGF expression*						
Negative	33	82	0.16	7	43	0.52
Positive	70	69		46	30	

a Overall survival at 2 years, logistic regression. VEGF=vascular endothelial growth factor.

, patients whose tumours did not show p53 expression had a higher probability of overall survival than those whose tumours presented p53 expression (*P*=0.01). By contrast, MVD and VEGF expression did not correlate with overall survival. Finally, neither MVD nor p53 and VEGF expression predicted survival in patients who did not receive chemotherapy (*n*=53).

## DISCUSSION

Surgical resection is the mainstay of treatment for gastric cancer. However, the prognosis after resection has remained unsatisfactory because of a high incidence of postoperative recurrence. The identification of variables in gastric tumour biology might lead to a more precise assessment of outcome and response to therapy. In the present study, we investigated the potential prognostic value of MVD, p53 expression, and VEGF expression.

p53 expression was detected in 46% of patients, which falls within the range (40–60%) of previously published gastric cancer series (Joypaul *et al*, 1994; Gabbert *et al*, 1995; Victorzon *et al*, 1996). Use of the immunohistochemical detection of p53 as a prognostic marker has yielded conflicting results (Fenoglio-Preiser *et al*, 2003). These discrepancies may, in part, be due to the limitations of p53 immunodetection. In fact, p53 immunoreactivity correlates with the presence or absence of gene mutations examined by direct sequencing in 50% of advanced gastric cancers when exons 5–9 are examined (Tolbert *et al*, 1999). Technical issues can also contribute to divergent results. In order to overcome these limitations, we followed a meticulous methodology using monoclonal antibody BP53-12 that is similar to antibody DO7, antigen retrieval, and a standard counting method.

In some studies, the expression of p53 was associated with a higher frequency of serosal, vascular, and lymphatic invasion, increased lymph node metastases and, consequently, a more advanced tumour stage (Kakeji *et al*, 1993; Kim *et al*, 1997; Monig *et al*, 1997; Maehara *et al*, 1999). By contrast, other authors have not shown the association of p53 expression with node metastases or serosal invasion (Baba *et al*, 1998; Maeda *et al*, 1998). Our study agrees with these latter results, since p53 expression did not correlate to a more advanced tumour stage. In agreement with these results, p53 mutations have been found in 37% of patients with early gastric cancer (Uchino *et al*, 1993), and p53 expression has even been detected in metaplastic gastric mucosa (Ochiai *et al*, 1996). Thus, p53 inactivation may occur at an early stage of gastric carcinogenesis, which explains our finding that p53 expression indicated a poor prognosis independent of the lymph node ratio or the depth of invasion (pT). Finally, the worse prognosis of patients whose tumours expressed p53 may actually reflect a higher tendency to recur. In our study, the recurrence rate in p53-negative patients was 28% compared to 44% in p53 positive patients (*P*<0.05). Similar findings have been reported in Japanese series (Maeda *et al*, 1996; Maehara *et al*, 1999).

The prognostic role of tumour angiogenesis was also investigated following a methodology accepted internationally (Vermeulen *et al*, 1996). We used a monoclonal antibody against adhesion molecule CD34 to stain the vascular endothelium, which was found to be superior to other markers (Tanigawa *et al*, 1996; Tanigawa *et al*, 1998). Our study confirm's the results of (Maeda *et al*, 1995) and (Xiangming *et al*, 1998) showing that MVD correlates with lymph node metastases in gastric cancer, as in esophageal cancer (Igarashi *et al*, 1998). MVD was also associated with a higher recurrence rate and a shorter disease-free survival in the univariate analysis. However, this association was not statistically significant in the multivariate regression analysis probably due to the positive correlation between MVD and both p53 expression and VEGF expression. The predictive value of MVD has also been suggested in other studies in which a higher MVD was associated with the development of distant metastases (Tanigawa *et al*, 1996) or bone marrow micrometastases (Maehara *et al*, 1998). Similarly, experimental studies using antiangiogenic agents, such as TNP-470, have shown effectiveness in decreasing tumour proliferative activity and inhibiting the development of liver and peritoneal metastases (Kanai *et al*, 1997; Yoshikawa *et al*, 2000). These findings reinforce the relationship between tumour angiogenesis and the spread of metastases.

Tumour angiogenesis is a complex, highly regulated process depending on the balance between activator and inhibitor factors (Castells and Rustgi, 2003). Vascular endothelial growth factor is a powerful and specific inducer of new vasculature in several neoplasms. In our study, MVD correlated with VEGF expression in tumour cells, in accordance with Maeda *et al* (1996). We used a standardised method to detect VEGF expression. Our patients were classified into two categories, by the presence or absence of staining reaction. We decided to consider cases as positives or negatives independent of the intensity of the staining. This fact can justify the relatively low proportion of patients whose tumours were considered negative for VEGF (25.6%) in the present study, which is similar to that recently reported in other series (Song *et al*, 2002; Joo *et al*, 2003).

Vascular endothelial growth factor induces the formation of new immature vessels, with basal membrane holes, favouring the progression of tumour cells into the vascular space. VEGF expression in gastric cancer has been associated with various clinicopathological parameters such as degree of differentiation (Tanigawa *et al*, 1997), intestinal-type tumours (Takahashi *et al*, 1996), lymphatic invasion, and vascular invasion (Maeda *et al*, 1996). Maehara *et al* (2000a) demonstrated that VEGF expression was an independent risk factor for vascular invasion that might account for a large number of metastases. This correlation was not observed in our series, but it should be taken into account that it corresponded to curatively resected patients who had a lower probability of metastatic dissemination. However, although VEGF expression was not associated with any tumour-related characteristic in our study, similar as in the series of Baba *et al* (1998), this parameter had an independent predictive value with respect to tumour recurrence, disease-free survival, and overall survival. These results suggest that VEGF expression might be a useful and powerful prognostic marker in patients with gastric cancer operated on for cure.

In the multivariate analysis, p53 expression and VEGF expression were independent prognostic factors. Similarly, concurrence of p53 expression and VEGF expression occurred in only 38% of patients, and there was no correlation between both markers. Our results indicate that p53 and VEGF expression are important factors to upregulate tumour angiogenesis. Whereas the role of VEGF as an inductor of angiogenesis is well known, the mechanism of p53 to do so is not as well established. A few experimental studies have been published linking p53 and angiogenesis. Kieser *et al* (1994) demonstrated that mutant p53 potentiates protein kinase C induction of VEGF, then promoting the development of new vasculature. Supporting these findings, Mukhopadhyay *et al* (1995) showed that wild-type p53 downregulated endogenous VEGF mRNA level, as well as VEGF promoter activity, whereas mutant forms of p53 had no effect. The authors suggested that wild-type p53 may play a role in suppressing angiogenesis. These experimental results contrast with those observed in clinical series. In fact, our results suggest that p53 and VEGF regulate tumour angiogenesis in patients with gastric cancer, but they do not support the fact that p53 stimulates the appearance of new vasculature by enhancing the expression of VEGF. Reviewing the literature, we found three clinical studies with similar findings as ours, confirming that the expression of p53 does not correlate with VEGF expression in gastric cancer patients (Baba *et al*, 1998; Giatromanolaki *et al*, 2000; Joo *et al*, 2002).

Preliminary studies suggested that the determination of p53 status and angiogenesis may be useful to predict response to chemotherapy. Indeed, p53 gene inactivation by either mutation or deletion often results in resistance to antineoplastic drugs. In these *in vitro* studies, gastric and esophageal cancer cells with p53 expression were resistant to 5-fluorouracil, mitomycin-C, and cisplatin (Lowe *et al*, 1994; Nabeya *et al*, 1995). Two clinical studies have evaluated the influence of p53 status in patients with locally advanced gastric cancer receiving neoadjuvant treatment. In one of them, patients with negative p53 expression had a greater tumour response to chemotherapy using 5-fluorouracil (72 *vs* 12%, *P*<0.004) (Cascinu *et al*, 1998). Similarly, the second study found that p53-negative and VEGF-positive patients responded better to chemotherapy with 5-fluorouracil and cisplatin (Boku *et al*, 1998). Finally, Diez *et al* (2000) reported in a series of 46 patients with gastric cancer receiving adjuvant chemotherapy that the absence of p53 overexpression was associated with longer survival. In agreement with these observations, we demonstrated that chemotherapy was less effective in patients whose tumours showed p53 expression, whereas MVD and VEGF expression did not have any predictive value in these settings. To our knowledge, this is the largest series assessing the relationship of p53 alteration to clinical outcome following adjuvant chemotherapy in gastric cancer, and its results may have noteworthy clinical implications.

In conclusion, this study shows that the expression of p53, VEGF, and a higher MVD are associated with tumour recurrence in gastric cancer patients resected with curative intent. The expression of p53 protein and VEGF are independent prognostic factors of disease-free and overall survival in gastric cancer patients having resection with curative intent. When tumour cells express p53 protein, the therapeutic efficacy of adjuvant chemotherapy is lost. Further studies are required to evaluate the potential clinical applications in the management of patients with gastric cancer.
